# Relationship between depression and olfactory sensory function: a review

**DOI:** 10.1093/chemse/bjab044

**Published:** 2021-10-07

**Authors:** Anna Athanassi, Romane Dorado Doncel, Kevin G Bath, Nathalie Mandairon

**Affiliations:** 1 INSERM, U1028; Centre National de la Recherche Scientific, UMR5292; Lyon Neuroscience Research Centre, Neuroplasticity and Neuropathology of Olfactory Perception Team, University Lyon, University Lyon 1, F-69000, France; 2 Division of Developmental Neuroscience, New York State Psychiatric Institute/Research Foundation for Mental Hygiene, 1051 Riverside Drive, New York, NY, 10032, USA; 3 Department of Psychiatry, Columbia University Medical College, New York, NY, 10032, USA

**Keywords:** olfactory perception, depression, humans, rodent models

## Abstract

Links between olfactory sensory function and effect have been well established. A robust literature exists in both humans and animals showing that disrupting olfaction sensory function can elicit disordered mood state, including serve as a model of depression. Despite this, considerably less is known regarding the directionality and neural basis of this relationship, e.g. whether disruptions in sensory function precede and contribute to altered mood or if altered mood state precipitates changes in olfactory perception. Further, the neural basis of altered olfactory function in depression remains unclear. In conjunction with clinical studies, animal models represent a valuable tool to understand the relationship between altered mood and olfactory sensory function. Here, we review the relevant literature assessing olfactory performance in depression in humans and in rodent models of depressive-like behavioral states. Rodents allow for detailed characterization of alterations in olfactory perception, manipulation of experiential events that elicit depressive-like phenotypes, and allow for interrogation of potential predictive markers of disease and the cellular basis of olfactory impairments associated with depressive-like phenotypes. We synthesize these findings to identify paths forward to investigate and understand the complex interplay between depression and olfactory sensory function.

## Introduction

Approximately 15% of the worldwide burden of disease is attributed to mental disorders (Prince et al. 2007; Atanasova et al. 2008), and 8 to 12% of the world’s population will be affected by major depressive disorder (MDD) at least once in their life (Weissman et al. 1996; Andrade et al. 2003). MDD is one of the most common neuropsychiatric disorders and represents a major public health concern. More than 264 million people of all ages are affected by MDD (WHO 2017). When focusing on epidemiological factors such as sex, research consistently reports greater prevalence of MDD in women, with female-to-male risk ratios of roughly 2:1 (Hirschfeld and Cross 1982; Kessler 2003; Gobinath et al. 2014; Kendler et al. 2020). MDD is associated with significant morbidity and mortality (Carney et al. 2002), with about 50% of depressed patients showing suicidal ideation or thought and 800,000 attempting and completing suicide every year.

MDD is a complex disorder that has at its core either a persistent depressed mood state and/or anhedonia (loss of interest and/or pleasure). Paired with one or both of these core features, a number of other symptoms must also be present, and can include effects on how an individual feels, thinks, eats, sleeps, or behaves. MDD is also associated with emotional, sensory, and physical problems (sadness, hopeless feelings, anxiety, moving/speaking more slowly, lack of energy) (WHO 2020), has multiple precipitating factors, and can have a multifaceted etiology. Identified risk factors include biological, genetic, epigenetic, and environmental variables (including hormonal fluctuations, genetic mutation, family history, and stress, to name a few) (Rochet et al. 2018; Kendler et al. 2020). Complicating the study of depression is the existence of differing subtypes and forms of pathology that include a depressive-like state (e.g. MDD, bipolar depression, seasonal affective disorder (SAD), to name a subset). Further, presentation of disease and risk factors contributing to the development of depression differ over the lifespan (Fiske et al. 2009; Schaakxs et al. 2017; Coleman et al. 2020). The diversity of causes, presentation, and evolution of depressive illness with aging has contributed to difficulty in studying the cause of disorder and the neurobiological underpinnings of pathology. Curiously, many of the more common symptoms of depression appear to either interact with or have direct effects on the sensory experience of the individual, which has the potential to provide novel insights into this complex disorder.

Several studies have highlighted a significant interaction between depression and the processing of signals in multiple sensory domains, with a primary focus on visual or auditory modalities (Kahkonen et al. 2007). The chemical senses (olfaction and taste) have also received attention in depression research and may provide some of the most interesting insights into the linkage between sensory function and mood. Olfaction is unique from other sensory modalities, in that olfactory inputs largely bypass thalamic relays and have nearly direct projections to brain centers that are implicated in emotional regulation and possibly mood state (Soudry et al. 2011). This direct access to limbic brain centers may be a vestige of the evolutionary importance of chemosensory function for the detection and response to threat, reproductive opportunities, and approach/avoidance of food sources. Thus, olfaction may be unique with regard to its direct access to neural substrates regulating vigilance and may be well situated to be involved in the regulation of mood.

Supporting a close linkage between olfaction and mood, the ablation or diminishment of olfactory sensory function can induce behavioral profiles reminiscent of depressive-like states in both rodents and humans (Atanasova et al. 2008; Croy et al. 2013; Taalman et al. 2017; Rochet et al. 2018). Further, greater severity of reported depressive symptoms has been associated with a greater severity of olfactory deficits. In those models, depressive symptoms can be partially rescued through olfactory enrichment (Lehrner et al., 2005; Brand and Schaal 2017; Rochet et al. 2018; Ballanger et al. 2019). While diminished olfactory sensory experience can promote the development of depressive-like phenotypes in model systems, questions remain as to whether the relationship between depression and olfactory sensory function is unidirectional. For example, does depressed mood state contribute to altered sensory function and drive decrement in sensory experience or does the decrement in sensory function worsen mood state? Studies assessing the impact of depression itself on olfactory function have been far less common leaving this a largely open question (Pollatos et al. 2007; Croy et al. 2014; Khil et al. 2016; Kohli et al. 2016; Croy and Hummel 2017; Pabel et al. 2018). Further complicating these studies may be a selection bias, with individuals in the general population having (but being unaware of) a compromised sense of smell or taste or failing to present at the clinic for sensory deficits alone (Oleszkiewicz et al. 2020). Thus, the incidence of olfactory deficits in the general population may be under-reported. This has the potential to skew our perception of the degree to which olfaction and mood are interrelated. Further, olfactory disturbance is present in several neurological conditions that have increased risk for depression as a comorbid feature (including Alzheimer’s, Parkinson’s, and Schizophrenia) (Schiffman 1997; Kovacs et al. 2003; Luzzi et al. 2007; Taalman et al. 2017; Carnemolla et al. 2020; Eek et al. 2021; Son et al. 2021). Olfactory impairment has also been reported in neurodevelopmental diseases, including autism spectrum disorder (Koehler et al. 2018), and has dramatically increased worldwide in the last year due to the COVID-19 infection (Pierron et al. 2020). Given these complexities, it may be difficult to disentangle effects observed in these varied populations that are related to depressive pathology from those due to neurobiological effects associated with other forms of diseases. For those studies that have begun to probe links between olfaction and mood, it remains to be determined if olfactory impairments in depressed patients are traits (persistent characteristics that were present before and after symptomatic remission) or states (characteristics that are only present during the symptomatic phase and disappear after treatment of depression). The goal of the following sections is to review the literature from human and animal models and attempt to clarify ways in which depression and olfactory performance may be related. For the purpose of clarity, we have tried to restrict our discussion to work in populations with major depressive disorder (MDD).

## Alterations in olfactory performance among humans diagnosed with depression

In studies that have assessed olfactory sensory function in populations of individuals suffering from MDD, methodological differences between studies have made results difficult to interpret or to relate across studies. To truly understand the effects of depression on olfactory sensory function, attention must be paid to multiple parameters. These include the assessment of olfactory threshold, discrimination, identity, familiarity, and the hedonic value of odors. Further, methodological considerations must also be taken into account, including the characteristics of the cohort (sex, disease history, precipitating factors contributing to depression, genetic risk, etc.), means of collecting olfactory measures (self-report, clinical assessment, etc.), and control of the intensity, complexity, or hedonic value of the odors being tested. Variation in the choice of the above-mentioned variables has made it difficult to arrive at a consensus with regard to the effects of depression on olfactory sensory function (Taalman et al. 2017). In the following sections, we review the data on olfactory function across a variety of these domains in patient populations suffering from depression.

### Evaluation of perceptual and cognitive performance in depression

A number of studies have been undertaken to assess olfactory perceptual function in populations suffering from depression. A meta-analysis of that work, from Rochet et al in 2018, provides a summary of the results for olfactory threshold, identification, intensity, discrimination, and hedonics to evaluate their potential as marker tasks for depression. In the majority of studies looked at, no association was found between depression (mainly MDD) and olfactory discrimination (Pause et al. 2003; Atanasova et al. 2010; Clepce et al. 2010; Negoias et al. 2010). Regarding odorant identification, most studies found that this dimension was not altered by MDD (Serby et al. 1990; Lombion-Pouthier et al. 2006; Pentzek et al. 2007; Scinska et al. 2008; Swiecicki et al. 2009; Negoias et al. 2010) with an exception (Zucco and Bollini 2011). In the study by Zucco and Bollini, depression was associated with a lower identification capacity. Further, the authors compared mild and severe cases of major depression and found that when the disease was more severe, deficit in identification was greater. For detection threshold, some studies identified an increased threshold to detect odorants in depressed patients (Pause et al. 2001; Postolache et al. 2002; Lombion-Pouthier et al. 2006; Negoias et al. 2010). In these studies, olfactory detection was assessed using four different olfactory tests, the single or two-alternative staircase detection procedure, the European Test of Olfactory Capabilities, and the “Sniffin’ Sticks” test battery. For 3 of the studies, MDD classification was based on a Beck Depression Inventory (BDI) score between 23 and 29. In the fourth study (Postolache et al. 2002), the patients suffered from seasonal affective disorder. Despite those positive results, additional studies have reported no relationship between depression and detection threshold (Scinska et al. 2008; Swiecicki et al. 2009; Croy et al. 2014). The discrepancies between studies may have arisen from a number of differences, including differences in the age or gender of the population being studied. For example, the study by Scinska et al. was comprised of older adults, and the work by Croy et al. assessed performance in only female subjects. Finally, the report by Swiecicki et al. included both patients suffering from uniporal and bipolar depressive disorder.

The aforementioned studies that failed to find an association between depression and olfactory detection threshold may represent true null results (true identification of no effects of depression on sensory function). Alternatively, difficulty in detecting group differences could have been influenced by heterogeneity in the sample being studied. Depression represents a grouping of multiple etiologies that all cluster under the umbrella of a single disorder. As there are multiple contributing factors to disease development and presentation of depression, specific disruptions to olfactory function may be unique to a given subgroup of individuals within the broader classification of the depressed population. To test this prediction, it would be important to take into account the specific profile of symptoms of depression (e.g. to attempt to control for symptom clusters, number and duration of episode(s), treatment history, age, and sex of the subjects, and family history). Additional key variables that may influence unique aspects of olfactory function include hormonal or age influences on sensitivity as well as treatment history on discrimination, among others. By sampling across different subtypes of depression, the ability to observe an effect on a specific dimension of olfactory sensory function in prior studies may have been diluted by the presence of multiple subgroups that were not affected on that dimension. Identification of unique effects on olfactory function in subgroups of depression (should they exist) may provide important insights into the neurobiological underpinnings of olfactory disturbance and its relation to depressive symptoms and risk factors for disease development. For instance, MDD can be observed both with and without melancholic symptoms which themselves may impact the hedonic perception or rating of odorants (Clepce et al. 2010; Fletcher et al. 2015).

### Evaluation of hedonic responses to odors in depression

Given the prominence of anhedonia as one of the core symptoms of depression, more work has been carried out focusing on the relationship between MDD and hedonic processing of odorants than other dimensions of olfactory sensory function. The majority of studies have found differences between patients and controls in the hedonic valuation of select odorants (Pause et al. 2001; Lombion-Pouthier et al. 2006; Atanasova et al. 2010; Clepce et al. 2010; Naudin et al. 2012), with a smaller proportion of studies finding no effects on hedonic perception (Swiecicki et al. 2009). In most studies, unpleasant odorants were perceived as more unpleasant (negative alliesthesia described by Atasanova et al. 2010) and pleasant odorants were perceived less pleasant in depressed patients (anhedonia) (Atanasova et al. 2010). Interestingly, some depressed patients rated some odorants as more pleasant (Lombion-Pouthier et al. 2006). These included odorants that have been shown to have anxiolytic/relaxing effects in control populations, such as lavender and citral (Hatano et al. 2012; Agatonovic-Kustrin et al. 2020). The positive ratings may be the result of the anxiolytic features of these odorants, diminishing negative scores through their improvement of mood, or by decreasing comorbid anxiety in depressed populations (Hatano et al. 2012; Agatonovic-Kustrin et al. 2020). The fact that hedonic scoring of odorants may be influenced by the ability of the odor to directly interact with mood, the choice of odorants in experimental settings, and measurement of comorbid pathology, such as anxiety, will be important for gaining a better understanding of the effects of depression on this dimension of olfactory function (Pause et al. 2001; Lombion-Pouthier et al. 2006; Rochet et al. 2018).

In addition to heterogeneity in the composition of populations and types of odorants chosen for testing, there is variability in the approach used to assess sensory function. For example, to test hedonic valuation of odorants in depressed patients, some studies used complex mixtures of odorants, which may be closer to physiological and natural conditions of everyday life experiences (Naudin et al. 2012). As in the previously described studies, authors found an association between depression and the hedonic perception of these complex mixtures. More specifically, depressed patients had difficulty identifying pleasant odorants in a mixture while they had no difficulty in identifying unpleasant ones (Naudin et al. 2012). This is in line with the results presented by Atasanova et al. (2010), where patients had a lower identification capacity for only the pleasant component of a complex odor during depressive episodes (Atanasova et al. 2010). Moreover, pleasant and unpleasant ratings for components of a mixed stimulus had a greater separation in their ratings for subjects without depression compared to depressed patients. This result was present despite no difference in the perception of intensity of the odorants between control subjects and those suffering from depression. Together, these results indicate that hedonic perception may be altered in multiple ways: qualitative alteration (hedonic rating), hedonic discrimination (gestalt rating of mixtures), and quantitative alteration (magnitude of unpleasantness).

### Comparison of impairment pre and post antidepressant treatment

In addition to the comparison of control subjects with those with depression, some authors have focused on assessment of olfactory impairments before and after antidepressant treatments within depressed populations. In this work, successful treatment of depression was shown to rescue some of the observed deficits in olfactory function (Naudin et al. 2012; Croy et al. 2014; Yuan and Slotnick 2014; Taalman et al. 2017). Olfactory deficits (alteration of olfactory intensity, discrimination, and hedonic perception) in patients with MDD were sensitive to antidepressant treatment, or therapy, with improvement of olfactory scores after treatment and sometimes, a positive impact of the duration of the treatment (3 versus 6 weeks) on olfactory performance (Croy et al. 2014). It should also be noted that ~30% of depressed patients are resistant to medication/treatment, begging the question of whether comorbid alterations in sensory function are similarly insensitive to treatment. While correlations have been established between severity of symptoms and the degree of olfactory deficit, it will also be important to determine the impact of antidepressant treatment and the degree of symptom resolution on olfactory performance to better understand this relationship. Such studies may provide new insights into the mechanisms underlying altered sensory function as a disease endophenotype, and possible neurobiological alterations associated with MDD.

As alluded to above, differences in the subtype of depression may contribute to the disparate results observed in olfactory studies. Beyond symptom clusters, additional parameters of depression might impact olfactory function, including duration of illness. Pabel et al. (2018) found that while the severity of depression was not associated with disturbance in olfactory function, the course and duration of depressive episodes was. In fact, a greater duration of symptom expression and recurrent illness was correlated with diminished odor detection (diminished threshold) and poorer odor identification, with an interaction between course and duration (e.g. longer and more recurrent illness was associated with greater impairment) (Pabel et al. 2018).

### Considerations for studying the relationship of depression and olfaction

Based on studies described in the sections above, it is clear that additional work is needed to further understand the relationship between depression and olfaction. In future work, multiple key variables will need to be taken into account, including the potential disparate effects of differing etiologies of depression on olfaction, base rates of olfactory disturbance in the general population, and the methods employed to assay the multiple aspects of olfactory functioning in humans. Furthermore, the parameters contributing to disease development, sex, age and hormonal status of subjects, the course and duration of illness, and treatment history will also be important factors to consider when studying the relationship between depression and olfaction. However, in patient populations, there is often difficulty in obtaining the level of precision required to control for many of these variables. As a consequence, work in translational animal models has the potential to identify specific deficits that can then be more carefully targeted and tested in clinical populations. Below, we highlight some of the work being carried out specifically in rodent models of depressive-like behavior and the relevance of these models for understanding disturbance in olfactory sensory processing.

## Rodent models of depression

Below, we introduce several commonly used rodent models that are believed to recapitulate depressive-like phenotypes or to model genetic or environmental risk factors for depression and their impact on olfactory sensory function.

### Relevance of rodent models for depression

Given the prevalence and high rates of morbidity and mortality associated with depression, numerous studies have emerged over the years attempting to better understand the neurobiological basis of disease (Krishnan and Nestler 2008; Douglas and Porter 2009; Hamon and Blier 2013; Yang et al. 2019). Indeed, work in humans present a number of variables that are difficult to control for, including genetic variability, medication history, living conditions, age, sex, and the specific presentation of depressive symptoms used for exclusion/inclusion. Thus, to make progress in this area, many labs have turned to animal models that provide a means to examine both the neural circuitry as well as neurophysiological systems and molecular pathways underlying the pathophysiology of core symptoms of disease. In particular, rodent models enable precise control of genetic, environmental, and pharmacological variables that are often lacking in human studies, and permit approaching this important question through behavioral, neurobiological, or genetic models of risk for depressive-like behaviors.

The diagnosis of complex neuropsychiatric disorders such as depression is based on, and relies on, clinical observations and often phenomenological symptoms reported by the patient themselves. Thus, the use of rodent models has led to some skepticism with regard to their utility in modeling a complex disorder. It is indeed challenging to translate the complex diagnosis of a disorder in rodent models, partly because of the heterogeneity of depressive disorder and also due to the difficulty in assessing symptoms such as feelings of sadness and suicidal thoughts in rodents. Given this complexity, it is more common to use models with high construct validity (e.g. common genetic variant, environmental risk factors, and precipitating experiences) and to then rely upon endophenotyping approaches that model individual and biologically tractable features of the disorder, as opposed to attempting to recapitulate the full phenotype of disease.

A core example linking olfaction and depressive-like behavior in rodents was revealed after bilateral olfactory bulbectomy (Kelly et al. 1997; Song and Leonard 2005). After bulbectomy, rodents showed depressive-like behaviors, including decreased libido and deficits in long-term explicit memory (resembling loss of interest and cognitive deficits in depressive patients, respectively) and also a deficit in passive avoidance, a decrease in exploratory activity and motivational behavior (Hall and Macrides 1983; Lumia et al., 1992; Kelly et al. 1997; Antunes et al. 2016; Almeida et al. 2017). These symptoms were accompanied by physiological and neurochemical modifications such as changes in the functioning of cortical-hippocampal-amygdala circuit, 5-HT and dopamine dysfunction, reduction in the concentration of brain noradrenaline or affected neuroimmune function with an increase in circulating interleukin-1β and tumor necrosis factor-α and a reduction in lymphocyte counts (Kelly et al. 1997; Pistovcakova et al. 2008; Almeida et al. 2017) biomarkers/symptoms that are similar to those observed in MDD (Strawbridge et al. 2015).

For many years, olfactory bulbectomy in rats has been used as an experimental model of depression to study and predict how patients will respond to a given antidepressant (Kelly et al. 1997; Cryan et al. 1999; Song and Leonard 2005). This procedure was particularly useful to identify changes in behavior associated with endocrine, immune, and neurotransmitter systems in depressed patients following the loss of the sense of smell and to then identify antidepressant treatments that might reverse alterations in mood. This model was critical in establishing a link between disturbed olfactory sensory function and resulting risk for depressed mood (e.g. disturbing olfactory sensory function can increase risk for depression). However, given that this manipulation destroys a key brain region for olfactory function, it does not allow researchers to test if depressed mood state can alter olfactory sensory function (e.g. whether depression contributes to altered olfactory performance). To be able to test this, alternate animal models of depressive-like behavior were needed. These models were developed to recapitulate genetic and environmental disturbances that contribute to depressive-like behaviors, and importantly do not directly involve destroying regions of the brain critical for basic olfactory sensory function (as is the case in olfactory bulbectomy).

In humans, multiple environmental and genetic risk factors exist and contribute to risk for depression. Given this fact, it is important to study multiple animal model of depressive-like behavior, in which depressive-like behaviors are elicited in different ways (Soderlund and Lindskog 2018). Using a diversity of approaches has the potential to aid in our understanding of how each of these risk factors may impinge upon and contribute to altered olfactory sensory function in the context of pathology. Below, we introduce a subset of the model systems that have been developed to express depressive-like pathology, as well as introduce what is currently known about the impact of these manipulations on olfactory sensory functioning and neural structures involved in olfactory sensory processing.

### Rodent models of depressive-like behavior and the impact on olfactory sensory function

Based on the complex etiology of depression, rodent models of depressive-like behavior have been developed that elicit “symptoms” either by manipulating the environment to which they are exposed (e.g. acute or chronic stressor) or through genetic or pharmacological means, including exogenous administration of glucocorticoids and introduction of genetic mutations associated with depression in human populations (McGonagle and Kessler 1990; Caspi and Moffitt 2006; Chen et al. 2006; Suris et al. 2010; Uher and McGuffin 2010). Some of the most useful models allow investigation at multiple levels of analysis, including interrogation of neurophysiological changes, alterations in neural circuits, as well as impact on molecular and genetic targets that may underlie behavioral disturbance. That being said, many models provide important insights, but also have limitations with regard to the translational potential or generalizability of their findings. Here, we provide a brief overview of some of the more commonly used rodent models of depression and their key characteristics.

#### Rodent models of genetic risk for depression.

Genetically engineered models provide an interesting approach to assess the genetic risk factors for MDD. Among the gene candidates involved in elevated risk for depression, BDNF is particularly interesting since it is involved in neuronal survival, differentiation, and synaptic plasticity. A methionine (Met) substitution for valine (Val) at the codon 66 of BDNF (BDNF^Val66Met^), identified in humans, has been associated with risk for depression and a variety of other neuropsychiatric disorders (Pezawas et al. 2008). Mice have been developed to carry a synonymous single-nucleotide polymorphism in the BDNF gene (val66met) which has been shown to alter brain anatomy, memory, and increased anxiety-related behaviors that are not normalized by the antidepressant, fluoxetine (Chen et al. 2006). The Val66Met mutation in the BDNF gene disrupts activity dependent release of BDNF has been shown to impact the migration of cells through the rostral migratory stream to the olfactory bulb, ultimately diminishing the number of newly born granule cells in the adult olfactory bulb (Bath et al. 2008). The decrease in OB granule cell neurogenesis has been associated with impaired olfactory discrimination abilities in these mice in a spontaneous cross-habituation task (Bath et al. 2008). In these mice, no effects were observed on odor investigation, habituation, or olfactory detection thresholds, indicating a very specific deficit in olfactory sensory function. However, much work remains to be done to understand whether additional deficits exist in olfactory function in these mice.

Another candidate pathway associated with risk for depression is disruption in serotonin function. Numerous genetic models of depression have been based on manipulating serotonin, including the generation of 5-hydroxy tryptophan (5-HT) receptor knockout mouse lines (Lemonde et al. 2003; Neumeister et al. 2004). Studies of these mice have highlighted an increased sensitivity to stress while others have shown that deletion of the serotonin related gene (e.g. tryptophan hydroxylase- *tph2*) leads to increased immobility in forced swim test, indicative of a depressive-like phenotype (Savelieva et al. 2008; Mosienko et al. 2012). Loss-of-function mutation in tryptophan hydroxylase (*tph*), the rate-limiting enzyme in 5-HT biosynthesis, also appeared to cause pro-depressive effects in mice (Zhang et al. 2005). Tph2^-/-^ dams have been shown to exhibit significant deficits in maternal care and pup retrieval, however, those effects did not appear to be due to general deficits in odorant detection or crude levels of odorant discrimination, based on an odor cross-habituation assay (Angoa-Perez et al. 2014). Further, Tph^-/-^ mice were able to learn to perform a reversal learning task where odorants were used as the guiding cue (Carlson et al. 2016).

In addition to genetic risk factors, a number of environmental factors have been identified that increase the risk for developing depressive-like symptoms and have been modeled in rodents. While these models have been heavily utilized to understand the neurobiology of depressive-like symptoms and treatment response, few have assessed the impact of these manipulations on olfactory sensory function. Below, we highlight a few of the more commonly employed rodent models of depressive-like behavior and the small amount that is known about their impact on olfactory function.

#### Unpredictable chronic mild stress (UCMS).

A common and well-validated model to induce depressive-like behavior is UCMS (Willner 2017). Over a period of several weeks, animals receive a series of different stressors (i.e. food and water deprivation, wet bedding, cage tilt, etc) several times a day and at unpredictable time points, causing moderate levels of stress. This manipulation produces long-lasting effects and has been established as a model that leads to a persistent and long-lasting depressive-like state in rodents. Consequently, rodents exhibiting depressive-like behavior generally show a decrease in sucrose preference, an increase of intracranial self-stimulation threshold or a loss of weight (Mineur et al. 2006), behavioral profiles that are indicative of a decrease in reward sensitivity and the development of anhedonia. Sustained and unpredictable stress applied in the UCMS model may recapitulate the varied stressful life events that significantly elevate risk for disorder and depressive-like behavior in humans. In this model, a decrease in the number of olfactory receptors has been observed (Li et al. 2015) as well as reduced neurogenesis in the OB (Yang et al. 2011), and impaired olfactory discrimination (Hu et al. 2020). However, we are not aware of any studies assessing other aspects of olfactory behavioral performance following UCMS.

#### Learned helplessness.

Because hopelessness is considered a central symptom of depression, the learned helplessness (LH) paradigm is commonly used as an animal model of depressive-like behavior (Seligman 1972). In this model, the rodent is placed in a closed chamber without any possibility of escaping and then receives several electric shocks applied to their feet. Animals are then placed in another chamber with a grid floor and receive multiple mild shocks, but now with the possibility of escaping. Depressive-like behavior is measured by the likelihood and latency to escape the electrical shock in the new context (e.g. shock avoidance). “Depressed” rodents that were trained in the learned helplessness paradigm frequently exhibit a delayed escape time (Seligman and Peterson 2007), while severely depressed rodents do not escape the shock at all (Maier and Seligman 2016). LH is considered a strong model of depressive-like behavior as the unpredictability and the uncontrollability of the acute stressors create a hopeless, inescapable, and uncontrollable situation, eliciting a depressive-like response. This model is a valuable tool to illustrate the negative cognitive bias described in depression, where patients generally exhibit a negative view of events and interpret them as not controllable. Based on our review of the literature, we have not found any reports assessing the effect of LH on olfactory performance or olfactory circuitry.

#### Social defeat model.

The social defeat model is a variant of the LH model but employs social conflict as a stressor to create psychological and emotional stress (Toyoda 2017). This model is based on the repeated subordination of a mouse or rat by a dominant peer. The protocol consists of placing an intruder rodent in the home cage of a large dominant male. Under these conditions, the resident animal attacks the smaller intruder. After several physical attacks, and co-housing with the aggressor, the defeated rodent undergoes behavioral testing to test for increased expression of depressive-like behaviors (e.g. social avoidance, increased defensive behavior, and increased anxiety, decreased locomotor activity, sleep disturbances, alterations in body weight, impaired immune functions) (Toyoda 2017). The social defeat model produces physiological changes and behavioral symptoms that reflect the features of posttraumatic stress disorder (PTSD) and depression (Hammack et al. 2012; Conoscenti and Fanselow 2019). Indeed, inescapable acute threats used in social defeat (as well as LH) replicate exposure to traumatic events occurring in life (stressful events or situations of power imbalance like bullying and sexual harassment) which increase the risk for PTSD and depression. While many of these studies use social approach as a metric of learned fear and social avoidance, we are not aware of any studies that directly assess the impact of social defeat on olfactory sensory function, beyond willingness to approach a social signal. In some instances, subjugated mice even fail to approach a novel animal in a neutral environment. The lack of approach to a conspecific could either indicate an active avoidance of conspecifics due to increased anxiety or could reflect a diminished ability to either detect the chemical cues emitted by the conspecific or discriminate them from the odor of the aggressive animal that was encountered during social defeat.

#### Early life adversity model.

The early life adversity model is based on the idea that adverse events in early life may shape the behavioral and the biological phenotype of the offspring based on altered neurodevelopmental trajectories, ultimately resulting in behavioral phenotypes that approximate depression or psychosis (Syed and Nemeroff 2017). These models have provided evidence for increased risk for later life negative outcomes that may be the consequence of altered development. Forms of early life adversity have included models of early maternal separation (MS) (Demaestri et al. 2020) and limited nesting and bedding (Gallo et al. 2019; Goodwill et al. 2019), among other manipulations.

Early maternal separation (MS) is a procedure that exposes rodent pups to a 3-hour (sometimes 6-hour) daily maternal separation and isolation stress from postnatal days 4 to 11 (or 21). Once these rodents are adults, they generally exhibit deficits in learning and memory (Thomas et al. 2016), increased anxiety-like behavior, and depressive-like behavior (Jin et al. 2018; Demaestri et al. 2020). More specifically, MS rearing has been shown to affect males and females similarly in early milestone development, yet only males showed changes in stress physiology and anxiety-like outcomes (Demaestri et al. 2020). Further, changes have been observed at the biological level, such as an increase in corticosterone levels and a decrease in brain neurotrophic factor (BDNF) (Wang et al. 2015).

The limited bedding model (LB) is different from the maternal separation model in form but not timing (see (Demaestri et al. 2020)). It consists of transferring the dam and pups to novel housing conditions several days following birth and leaving the dam and pups in housing conditions with limited access to material resources for nest building for a period of seven days (Gallo et al. 2019). LB affects the timing of early developmental milestones, somatic growth, and stress physiology in both sexes, yet only female pups reared under these conditions showed later development of depressive-like behaviors (Goodwill et al. 2019). LB females, but not males, exhibited depressive-like behaviors on both traditional assays as well as newly developed home cage monitoring measures. These effects emerged during adolescence and became more severe in adulthood, mirroring the sex bias in risk, time course, and diversity of behavior features of pathology observed in humans. Further, LB effects could be rapidly rescued by ketamine, a fast-acting antidepressant (Goodwill et al. 2019).

Together, these data suggest that early life adversity like LB and MS can have a crucial impact on behavior during adulthood, causing debilitating long-lasting consequences such as elevated risk for depressive-like behavior. Preliminary data from our lab has also found that LB rearing leads to diminished ability to acquire learned discrimination of highly similar odorants on an olfactory perceptual learning task, as well as effects on olfactory bulb (OB) neurogenesis (unpublished). Further data on the effects of these manipulations on olfactory sensory function are lacking.

#### Corticosterone model.

Some models also recapitulate neurobiological alterations observed in MDD such as alterations in brain areas involved in stress-processing (i.e. increased activity in the prefrontal cortex, the amygdala, the hippocampus, the nucleus accumbens, and the habenula), in the stress axis (i.e. dysregulation of the hypothalamic-pituitary-adrenal axis) and in the immune system (i.e. neuroinflammation) (Brites and Fernandes 2015; Zang et al. 2018; Ceruso et al. 2020). For instance, chronic exposure to elevated levels of corticosterone (i.e. injection of a synthetic form of the hormone corticosterone) mimics chronic stress and evokes a depressive-like state in animals. Several studies have shown that corticosterone-injected rodents exhibit behavioral changes indicative of a depressive-like phenotype, including increased immobility in the forced swim test, decreased grooming and elevated anxiety-like behavior in the Open field and Light/Dark-Box tests, as well as anhedonia with a decreased sucrose preference (Baez and Volosin 1994; Mitra and Sapolsky 2008; Dieterich et al. 2019). In this model, the authors evaluated olfactory discrimination using an automated operant conditioning procedure and revealed alterations in the fine discrimination of highly similar odorants with no effects on the discrimination of highly dissimilar odorants. In addition, pronounced deficits in olfactory acuity (ability to discriminate between highly similar odorants) and olfactory memory have been also observed in this model and rescued by the antidepressant fluoxetine (Siopi et al. 2016).

In summary, while a number of genetic and environmental risk factors have been shown to contribute to the development of depressive-like behaviors, few have assessed the impact of these manipulations on olfactory sensory function. Given the diversity of models and factors that contribute to the development of depressive-like phenotypes, it seems that this would provide an excellent opportunity to investigate the impact of differing antecedents or risk factors for depression on risk for olfactory disturbance, and possible neural underpinnings driving those effects.

## Neural basis of olfactory impairments in depression

It is assumed that the existence of a link between olfaction and depression would be due to effects on anatomical structures that are common to both olfactory and emotional processing (amygdala, hippocampus, orbitofrontal cortex, prefrontal cortex, accumbens nucleus, parahippocampal gyrus, insula, ventral striatum). The overlap in neural structures mediating stress responding and processing of olfactory signals suggests that olfactory structures could be involved in emotional disturbance, and could explain why depression is often accompanied by alterations in olfactory function (Croy and Hummel 2017). In addition, olfactory and limbic systems are considered “evolutionarily old” systems which have co-evolved together. Thus, olfaction which is likely used to sense mates, danger, and food sources could have a strong role in regulating motivation and by proxy emotional state and behavior (Croy and Hummel 2017). In the following sections, we review anatomical effects associated with depression in humans that may explain comorbid alterations in olfactory processing.

### Impact of depression on neural circuits implicated in olfactory processing in humans

Odor perception is the result of odorant molecules binding to olfactory receptors located on sensory neurons in the nasal olfactory epithelium. These neurons then project to the OB, followed by projection to secondary olfactory areas including the anterior olfactory nucleus, olfactory tubercle, entorhinal cortex, piriform cortex, and cortical amygdala (Shepherd 1972; Wilson and Sullivan 2011). The signal is then further transmitted to additional brain centers including the hippocampus, thalamus, the ventral anterior insula, the orbitofrontal cortex (OFC), and the ventral tegmental area (Shepherd 1972; Wilson and Sullivan 2011; Courtiol and Wilson 2015). Thus, the olfactory system appears to have near-direct access to both cortical centers for perceptual processing as well as brain regions implicated in emotion and processing of reward.

Anatomical impairments of olfactory structures after depressive episodes have been observed in humans. It has been shown using magnetic resonance imaging (MRI) that depression leads to a decrease in OB volume and that the degree of OB volume reduction is correlated with the severity and maintenance of the disease- greater reduction is associated with greater impairment (Croy and Hummel 2017). Thus, the size of the OB could constitute a first biomarker for enhanced vulnerability to depression (Negoias et al. 2010).

A decrease in hippocampus, cingulate cortex, and habenula volumes has also been observed in depression (Campbell et al. 2004; van Tol et al. 2010). Moreover, some nuclei of the amygdala appeared to be predisposed to atrophy in depressed patients (Yao et al. 2020).

Using functional imaging in humans (fMRI), depression has also been associated with modified activity in other olfactory structures. For example, hyperactivity of the amygdala has been observed in depressed patients compared to controls (Drevets 2003). In the OFC, two subregions have been shown to be differentially engaged during depression: the ventromedial OFC is involved in anxiety and rumination and has shown to be hyperactive in depression while the dorsal OFC, which is involved in attention and working memory, has been shown to be hypoactive in depression (Rochet et al. 2018). Moreover, researches have found hyperactivity of the insula in depressed patients during resting state imaging and have shown that increased activity of this structure is associated with increased rumination in depression (Sliz and Hayley 2012). Finally, in accordance with studies of depression using animal models, human imaging studies have shown hyperactivity in the habenular region linked to symptoms of depression (Ranft et al. 2010). Different neuroimaging studies revealed changes in neural activity in these brain areas in response to odorant stimulation or autobiographical odor memory (Zatorre et al. 1992; Sobel et al. 1998; Rolls 2004; Rochet et al. 2018) suggesting that the observed alteration in activity and morphometry of these regions in depressed populations could impact olfactory function.

The hippocampus and the amygdala, which receive dense projections carrying information from the olfactory system, play a key role in the regulation of emotional learning and memory (Soudry et al. 2011). These structures have been reliably shown to be affected in depression (Yao et al. 2020) which could explain some alterations in the cognitive aspects of olfactory perception, including identification, olfactory learning, or memory during depression (Lemogne et al. 2006). Depression can also have a strong impact on sleep, leading to significant disturbance (Agargun et al. 1997) which has been associated with impairment in odor identification (Killgore and McBride 2006; Prehn-Kristensen et al. 2015), and cognitive function (including issues with memory, concentration, and arousal). Together, such effects may serve to alter odor perception.

To better understand the relationship between olfactory disturbances, and to determine if they may result from, contribute to, or are independent of other depressive symptoms, a more systematic evaluation of basic olfactory perception (detection, spontaneous discrimination, hedonic value, intensity) should be carried out and placed in the context of additional measures of cognitive functioning.

### Impact of depression on neural circuits associated with olfaction in animals

As mentioned previously, to further our understanding of the neural circuits involved in depression, many have turned to animal models. Here, we summarize the data related to altered neural networks in rodent models of depressive-like behavior that may contribute to disturbance in olfactory sensory function.

The UCMS model in rats has been linked with a reduction in volume of the OB, a disturbance in presynaptic function, and a reduction in the number of olfactory receptor neurons in olfactory epithelium (Li et al. 2015). In two regions of the brain, there are continued high levels of neurogenesis in adulthood, the OB and the hippocampus (Ming and Song 2011). While numerous studies have shown an association between reduced hippocampal neurogenesis and increased risk for depressive-like symptoms (Warner-Schmidt and Duman 2006; Eisch and Petrik 2012), fewer studies have tested the relationship between MDD and OB neurogenesis (Siopi et al. 2016). The reduction in levels of neurogenesis and OB volume in the UCMS rat model indicate that the same stress that contributes to the expression of depressive-like behavior can impact OB neurogenesis. While there is debate about the existence of adult OB neurogenesis in humans (Curtis et al. 2011; Sanai et al. 2011; Bergmann et al. 2015; Lim and Alvarez-Buylla 2016), numerous studies have revealed reduced OB volume and olfactory sensitivity in patients with MDD (Pause et al. 2001; Negoias et al. 2010) and the presence of neuronal turnover in the olfactory epithelium. Thus, it is possible that stress could contribute to impairments in olfactory perception in MDD patients, however, these linkages must be formally tested. While specific olfactory impairments have not yet been highlighted in animal models of depression, it would be relevant to test specific parameters, including olfactory acuity and sensitivity as well as discrimination ability in animal models of depression in which reduced OB neurogenesis has been observed. Importantly, studies showing decreased neurogenesis in animal model of depression have been able to restore levels of neurogenesis in the hippocampus (Banasr and Duman 2007; Rochet et al. 2018) as well as in the OB (Siopi et al. 2016) with treatment with antidepressants (fluoxetine), consistent with improvement in olfactory deficits following treatment.

To better understand the brain regions involved in olfactory deficits in models of depressive-like behavior, Croy and Hummel (2017) posit that olfactory receptor turnover rate in the olfactory epithelium is decreased in depression. This is a potentially interesting potential mechanism for olfactory disturbance in MDD and could explain impaired olfactory threshold, identification or discrimination, in patient populations (Croy and Hummel 2017). Other studies have shown that the habenula (involved in the transfer of olfactory information to other brain areas) might also be affected in depression. Models of bilateral bulbectomy in rodents revealed a higher level of apoptosis in the habenula during depression, which could contribute to its role in olfactory disturbance in depression (Brand and Schaal 2017).

Numerous studies using the LH rat model of depressive-like behavior have shown important metabolic changes in regions of frontal cortex and hippocampus as well as a reduction in BDNF in the medial prefrontal cortex and dentate gyrus, disturbance in lipid metabolism, glutamatergic metabolism, and neurotransmission (Shirayama et al. 2015; Dwivedi and Zhang 2016; Liu et al. 2018). LH was also observed to have significant impacts on the habenula, amygdala, insular and cingulate cortex (Shumake and Gonzalez-Lima 2003; Shumake et al. 2004). Interestingly, these regions are known to be involved in attention, emotional, and cognitive processes and also in olfactory processing (Kesner et al. 2002; Pouliot and Jones-Gotman 2008; Veldhuizen et al. 2010; Brand and Schaal 2017). In this context, alterations of these regions in LH rats may significantly diminish olfactory sensory function, however, olfactory performance has not yet been thoroughly tested in this model. Possible effects of disturbance in the function of these regions could include diminished olfactory sensitivity or altered hedonic perception of odorants, which have not yet been evaluated in LH rats.

Numerous studies have also analyzed the consequences of repeated exposure to social defeat in mice on neural activity in the prefrontal cortex, cingulate cortex, hippocampal formation, amygdala, and hypothalamic nuclei (Sheline et al. 2002; Videbech and Ravnkilde 2004; Bourne et al. 2013). As described above, these brain regions form important limbic structures implicated in a wide variety of emotional, cognitive, and behavioral control processes (Kumari et al. 2003; Surguladze et al. 2005; Sheline 2011), and have also been implicated in the development of PTSD. These regions have also been implicated in the processing of olfactory information. As one example, social defeat is associated with increased activity in the amygdala which could impact the processing of olfactory signals, influencing the perceived hedonic value, the discriminability, or emotional significance of olfactory stimuli. Hamilton & Gotlib showed in 2008 that depressed patients exhibited higher amygdala activity in response to negative (but not positive) emotional stimuli that they had memorized (Hamilton and Gotlib 2008). Based on that work, it seems relevant to test the effects of social defeat on hedonic perception and response to odors.

A recent study by Czarnabay et al. (2019) found that rodents that experienced maternal separation (MS) exhibited a significant delay in the proliferation and differentiation of neurons in the hippocampus and OB (Czarnabay et al. 2019). Further, MS pups took more time to identify odorants in an olfactory learning task. Thus, stress during a key period in early development appears to have negative and long-lasting effects on olfactory function in the offspring. While the primary results indicate deficits in olfactory memory of rodents raised in MS conditions, further experiments are needed to test for broader effects of MS on olfactory sensory function and possible mechanisms linking delayed development of major olfactory structures with deficit in olfactory memory.

In summary, studies using animal models of depressive-like behavior have commonly shown a reduced volume of crucial olfactory and limbic structures, including the OB and hippocampus, along with diminished neurogenesis. Numerous studies have also highlighted dysfunction of major neurotransmitter systems associated with metabolic changes including impacts on the serotonergic and dopaminergic systems in regions that are implicated in the sensing and perception of olfactory signals. While some studies have found links between depressive-like behavior and alterations in olfactory identification, few studies have tested the effects of models of depressive-like behavior on other parameters of olfactory perception. While depression has been linked with modified hedonic perception of odorants in humans, the linkage between depressive-like phenotypes and hedonic perception of odorants in rodent models remains largely unstudied. Studies focusing on olfactory parameters like odorant sensitivity, discrimination, and hedonic perception of odorants in animal models of depression are required to further our understanding of the links between olfaction and depression and to connect results observed in animal models with results found in depressed patients. In aggregate, these results illustrate the need for additional work and more thorough analysis of olfactory perception (in particular, hedonic processing of odorants) in the different animal models of depressive-like behavior. Specifically, there is a lack of important information with regard to results from animal models of depression and olfactory perceptual capacities, including effects on olfactory threshold, discrimination, identification, or hedonic perception that will be needed to draw clearer conclusions regarding the impact of depression on olfaction and its neural basis.

## Conclusion

With regard to the existing bidirectional relationship between olfaction and depression, it seems that the quality of life of depressed patients is more affected when alterations of the olfactory processing are observed (Kohli et al. 2016). Quality of life is a complex concept, influenced by the physical health of the subject, their psychological state, their level of independence, their social relationships, and their perception of their environment (Rochet et al. 2018). However, since odors provide critical information for survival (e.g. food safety and enjoyment for example) and guide our social relationships, behaviors, and mood (e.g. fear and happiness, motivational behaviors, food intake, reproduction, communication, transmission of anxiety etc.) (Brand and Schaal 2017; Croy and Hummel 2017), the impact of depression on olfactory function has the potential to impact or worsen the quality of life in the context of broader pathology. Studying the relationship between depression and olfactory sensory function has the potential to provide unique insights into the possible neurobiological basis of disease, symptom expression, and possible novel targets for treatments and interventions. However, in executing such studies, key variables must be taken into account when choosing the sample population, model system, and measures to assess these critical questions.

## Future Research

In the current review, we have discussed a number of topics related to our current understanding of the relationship between depression and olfactory sensory function in both human clinical populations and animals models of this debilitating disease. Based on our assessment of the literature, a number of issues remain to be tackled in this field. In clinical populations, there is a great deal of variability in both methods being used to test olfactory sensory function, dimensions of olfactory function being tested (sensitivity, discrimination, hedonic valuation), and heterogeneity in selection criteria for inclusion of individuals with varied forms of depression. In future work, it will be important to take care to assess multiple aspects of olfactory sensory function as well as control for the composition of the population (e.g. controlling for stressful life experiences, prior treatment, and genetic risk factors for depression, and rates of olfactory disturbance in the broader population). We have also highlighted the potential importance of using animal models to further probe these links. Specifically, more work is needed to assess sensory function at the neural and behavioral level in models of genetic and environmental risk for behavioral profiles associated with depressive-like behavior. The use of animal models may provide the level of control that is often difficult to attain in human samples to further assess the directionality of the relationship between depression and sensory disturbance. Further, such models provide researchers with the ability to directly probe the neural underpinnings of these effects to better understand the reason for the overlap between sensory disturbance and pathology. In summary, further studies will be critical in bridging the current gap in knowledge regarding the relationship between depression and olfactory sensory function and such work will provide greater insight into the neurobiological basis of depression.
